# A compact two elements MIMO antenna for 5G communication

**DOI:** 10.1038/s41598-022-07579-5

**Published:** 2022-03-04

**Authors:** Ashfaq Ahmad, Dong-you Choi, Sadiq Ullah

**Affiliations:** 1 Communication and Wave Propagation Laboratory, Department of Information and Communication Engineering, Chosun University, Gwangju, 61452 South Korea; 2 Department of Telecommunication Engineering, University of Engineering and Technology, Mardan, 23200 Pakistan

**Keywords:** Electrical and electronic engineering, Nanoscience and technology

## Abstract

This study presents a simple, miniaturized, and low-profile multiple-input multiple-output (MIMO) antenna operating at 29 GHz with reduced mutual coupling between the antenna elements for futuristic 5G communication. The proposed design employs two radiating elements with slits in the radiators to produce high isolation among the antenna radiators. The MIMO antenna maintains a compact structure of 11.4 × 5.3 mm^2^, which is the smallest size compared to previous 5G antennas. Roger’s 4350B laminate was employed as a substrate material. At 29 GHz, low mutual coupling of − 36 dB, low envelope correlation coefficient (ECC < 0.001), and high diversity gain (DG > 9.8 dB) are achieved. The proposed design is examined in terms of the S-parameters, diversity gain, radiation pattern, and envelope correlation. Compared to the straight antenna element, an improvement of − 20 dB is observed in the isolation for both the simulated and measured results.

## Introduction

With the increasing demand for high data rates, MIMO antennas have gained significant attention and are considered a key technology in millimeter-wave communication. MIMO technology is highly recommended owing to its promising features of enhanced data transmission speed, coverage area, and resistance to multiple paths fading^1,2^. However, mutual coupling due to surface wave propagation and inadequate isolation among the elements of a massive MIMO system causes poor impedance and radiation performance and degrades channel capacity^3^. Increasing the physical distance between the radiators is a simple manner to achieve high isolation, but it results in a large antenna size. Researchers have attempted to develop methods to maximize the packing density while enhancing the isolation between the radiators. Thus, optimizing mutual coupling would be significant in wireless communication systems.

A MIMO antenna incorporates minimum two radiating elements, placed at a specific distance to achieve minimum mutual coupling between them, but very limited space is available in front-end modern communication systems. Different approaches have been recommended by the researcher to reduced mutual coupling between the radiators. In^4^, a stub on the lower ground plane was used in perpendicularly placed radiators to achieve minimum mutual coupling. In^5^, more than 20 dB isolation is observed among the MIMO patch antennas by using near field resonator above each antenna elements. A tree-shaped MIMO antenna structure was proposed in^6^ with isolation > 16 dB over the entire ultrawide band. In^7^, isolation was increase by adjusting the structure of the ground plane under each resonator in order to make mutual coupling out-of-phase between the ground plane and free space which cause in isolation enhancement by more than 10 dB between the diagonal and adjacent elements. However, this approach increased the thickness of the antenna, reducing its wider applications in low-profile arrays. In^8^, six metal strips were incorporated between the radiators to efficiently reduce the mutual coupling without affecting the gain and radiation pattern. Another approach is to use slits in the ground plane, but it cause in backward radiation^9^. Similarly, mender line slots, L- and T-shaped ground branches were added to the ground plane resulting in high isolation at the cost of fabrication complexity^10–12^. In^13^, a novel parasitic decoupling technique is proposed having efficiency more than 64% for closely coupled antenna. Similarly a Wilkinson power divider is used as to enhance isolation between the radiators as shown in^14^.

Researchers have suggested remarkable techniques to reduced mutual coupling between the radiators, such as employing a defective ground plane^15^, electromagnetic bandgap structures^16^, and a perpendicular feeding network^17^. In^18^, parasitic F-shaped stubs were used between the radiating patches to achieve good isolation. An electric-LC resonator was used at two-element MIMO antenna for mutual coupling reduction^19^. In^20^, a Y-shaped stub ensued by rectangular slots in the ground plane was employed for better isolation between wrench MIMO antennas. In^21^, neutralization line is presented for the first time to achieve isolation enhancement. Each neutralization line consists of metal strip and in the middle of antenna elements a reactive component attached.

This study proposes a low-cost, low-profile, miniaturized two-element-based MIMO antenna system with significantly reduced mutual coupling of S21 < − 36 dB. The antenna elements are rotated at 45° in the clockwise direction, and two slots are introduced in the radiating part to achieve better isolation in these closely packed elements. These slots extend the physical separation between the resonators, resulting in high isolation^22^. The antenna has a simple structure which is excited using a 50-Ω feedline. The proposed design is employed on a Rogers 4350B laminate with a thickness of 0.8 mm. Satisfactory agreement between both the simulated and measured results was obtained, which emphasizes that the proposed design could be a prime candidate for 5G communication because of its low envelope correlation, high diversity gain, and low mutual coupling between the radiators.

## Antenna configuration

The proposed antenna elements are evolved from simple rectangular shape patch antenna. The detailed dimensions of proposed MIMO antenna are determined using the well-known transmission line theory^23^. The effective resonant length (*L*_*re*_) and width (*W*) for specific resonant frequency (f_r_) are calculated using mentioned theory^23^:

1 $$L_{re} = \frac{c}{ 2f_{r} \sqrt {\left[\frac{{\varepsilon_{r} + 1}}{2} + \frac{{\varepsilon_{r} - 1}}{2}\left( {1 + 12\frac{h}{W}} \right)^{ - 0.5} \right]}} - 2\Delta L$$

2 $$W=\frac{1}{2{f}_{r}\sqrt{{\mu }_{o}{\varepsilon }_{o}}}\sqrt{\frac{2}{{\varepsilon }_{r}+1}}$$

Similarly, *h* is the thickness of the substrate, c is the speed of light, ${f}_{r}$ is the resonance frequency, and $\Delta L$ represents the differtial change in length due to fringing. Furthermore, ${\varepsilon }_{o},{\varepsilon }_{r ,}$ and ${\mu }_{o}$ are the free space permittivity, relative permittivity, and permeability of free space, respectively. Using the above mentioned equations, dimensions of the presented antenna were obtained for 29 GHz.

**Figure 1:**
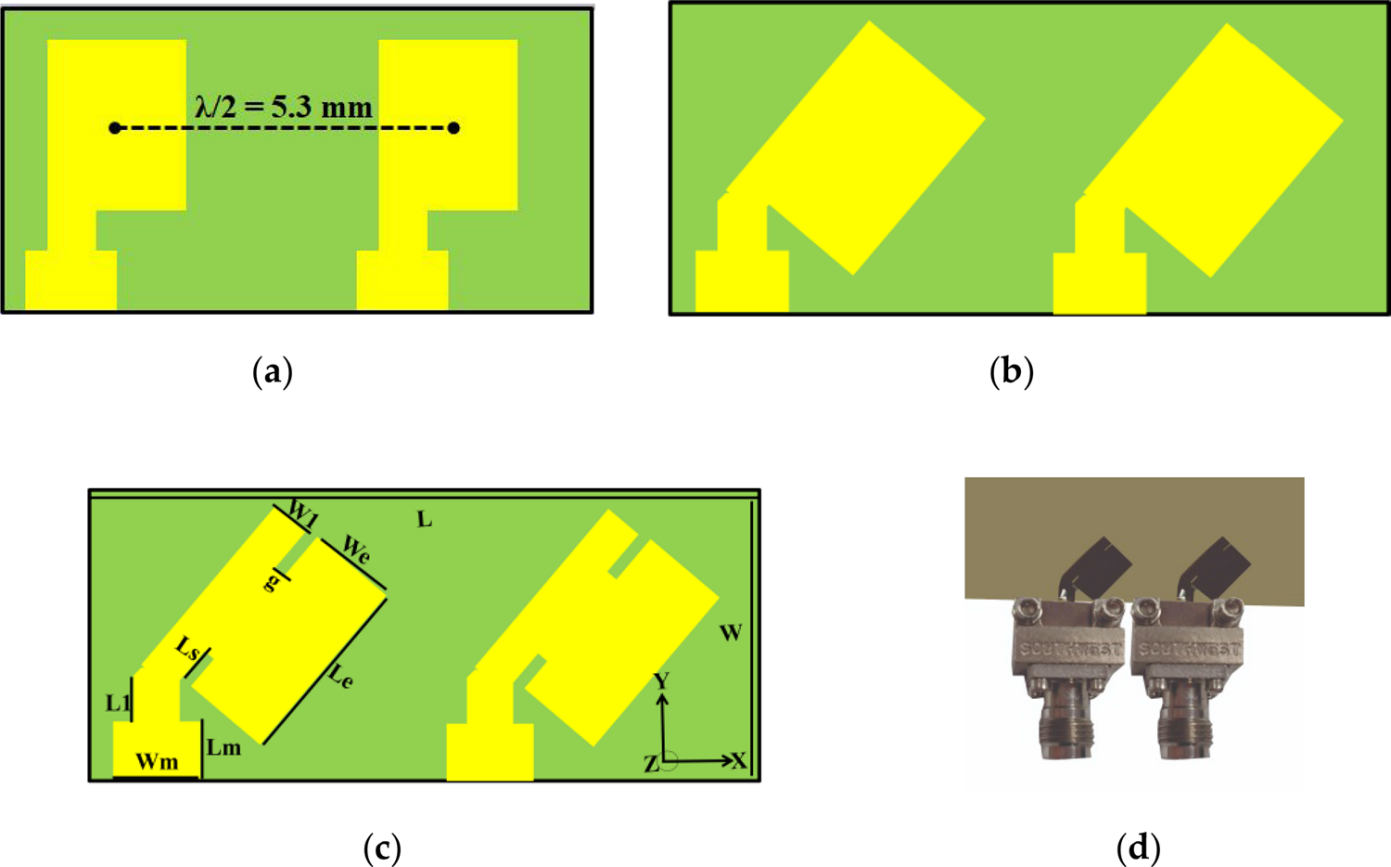
Design evolution of the proposed MIMO Antenna (**a**) Straight elements (**b**) Bend elements (**c**) Proposed (**d**) Prototype.

The design evolution of the two-element MIMO design is presented in Fig. 1. The proposed MIMO antenna has overall dimensions of 11.4 × 5.3 × 0.8 mm^3^, which occupies less volume compared to other antennas. Two radiating patches with slits, rotated at 45°, are printed on Roger 4350B substrate with 0.8 mm thickness and relative permittivity of 3.66. The length and width of the radiators were 3.62 mm and 2.7 mm, respectively. The substrate is backed by a common ground plane. Initially, straight antenna elements were designed, each operating at 36 GHz with mutual coupling S_21_ < − 16 dB. Low mutual coupling between the patches was observed by introducing slots and rotating them by 45° in the clockwise direction. Slots increased the current path, leading to mutual coupling reduction at the desired frequency of 29 GHz. The slots and stub (left part of a radiator) act as a capacitor and inductor, respectively, forming a parallel *LC*-equivalent circuit. Figure 1a, shows the straight elements with a center-to-center distance of half of the free space wavelength, i.e., 5.3 mm. Figure 1b, presents antenna elements rotated by 45° in the clockwise direction, while proposed design is shown in Fig. 1c; the fabricated prototype is shown in Fig. 1d.

The proposed MIMO design operates at 29 GHz with dimensions of *L* = 11.4, *W* = 5.3, *L*_*e*_ = 3.6, *W*_*e*_ = 1.8, *L*_*s*_ = *W*_*1*_ = 0.7 mm, *W*_*m*_ = 1.3, *L*_*m*_ = 1.2, *L*_*1*_ = 1.2, and *g* = 0.18 (all dimensions in mm). The antenna was modelled and optimized using the commercially available CST MW Studio Suite software.

## Results and discussion

**Figure 2:**
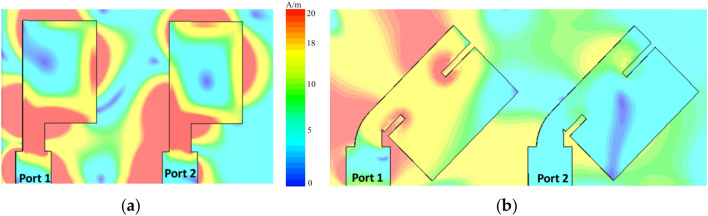
Surface current distribution at 29 GHz (**a**) Straight elements MIMO antenna and (**b**) proposed elements MIMO antenna.

The mutual coupling performance of the presented two elements MIMO antenna was analyzed by using the surface current distribution. Initially slots were introduced in the radiators and rotated by 45° in the clockwise direction. The current distribution on the surface of proposed design is shown in Fig. 2. When port 1 was excited, port 2 was terminated with a 50-Ω load. For the straight elements array, more coupling was observed between the two radiators, as shown in Fig. 2a. However, by introducing slots in the radiating elements and rotating each element by 45°, as shown in Fig. 2b, considerable reduction in surface current is observed, which resulted in losses mutual coupling with the neighboring patch.

**Figure 3:**
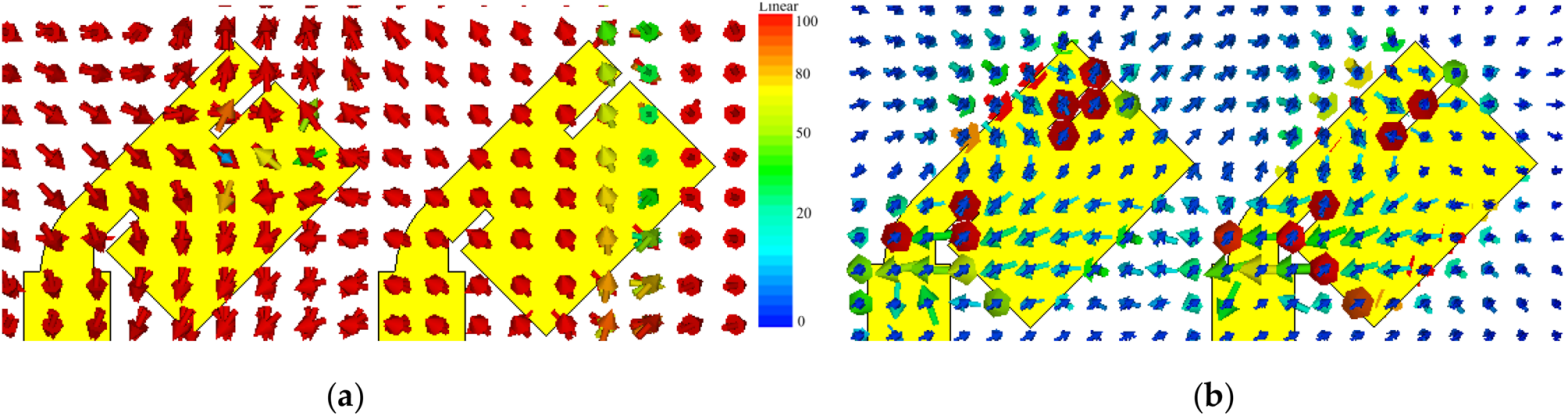
(**a**) E-field distribution and (**b**) H-field distribution of the reported MIMO antenna design at 29 GHz.

Figure 3a shows the simulated electric field distribution at 29 GHz. It is shown that antenna 1 is excited while antenna 2 is connected with 50-Ω load. It is evident from the electric field distribution that entire length of antenna patch is responsible for radiation. In the E-plane, the mutual coupling is in the TM mode. For further clarification, simulated magnetic field distribution at 29 GHz is presented in Fig. 3b. It can be observed that the high current densities could be found around the antenna slit which lead to low mutual coupling between the radiating elements. For magnetic fields, TE mode is observed. When the square patch antenna is excited from the diagonal, it has TM_01_ and TM_10_ modes with equal amplitude and phase.

**Figure 4:**
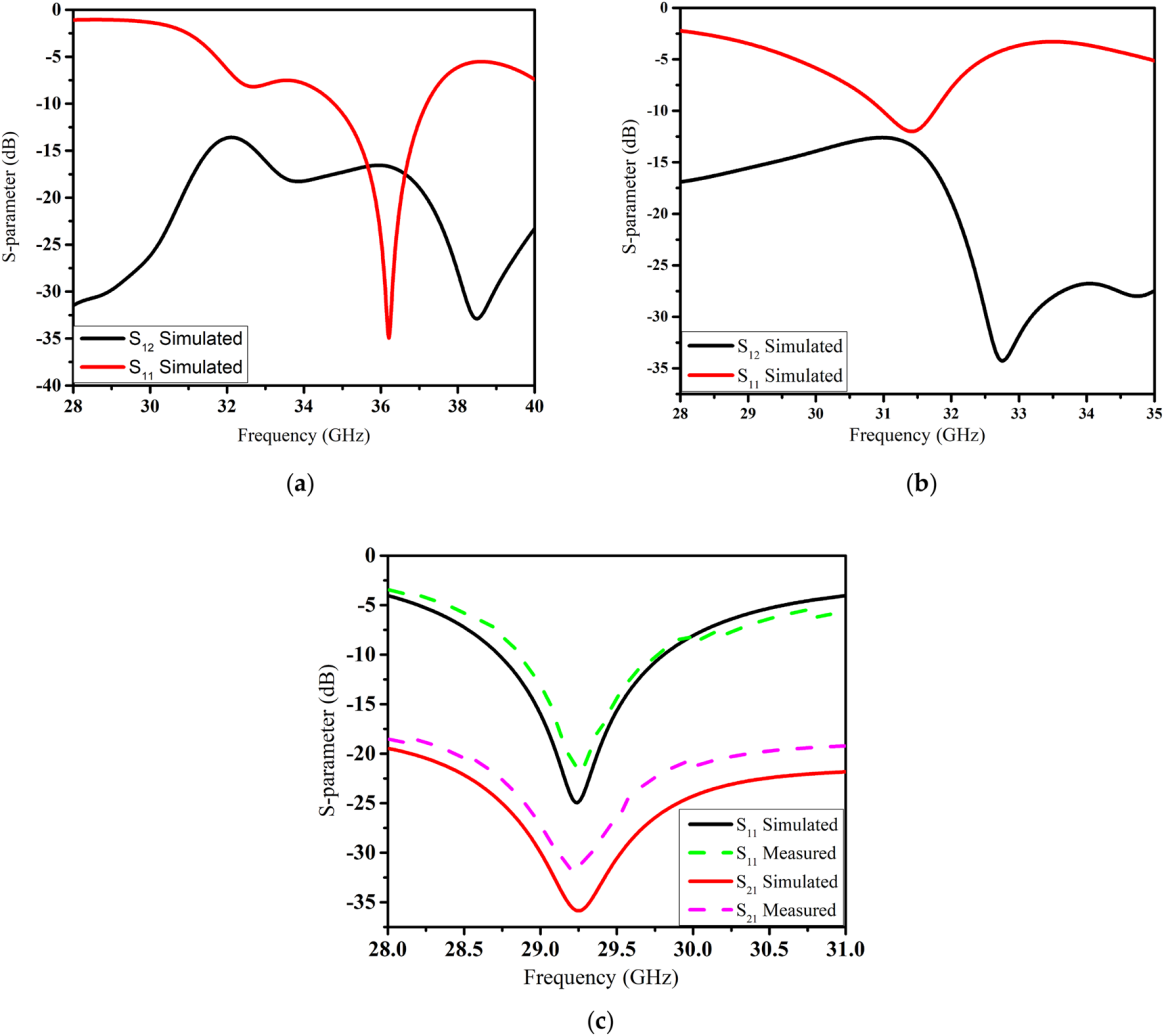
S-parameter of (**a**) straight elements (**b**) bend elements (**c**) and proposed elements MIMO antenna.

The scattering parameters of the straight elements MIMO antenna are shown in Fig. 4a. This array resonates at 36 GHz with a reflection coefficient (S_11_) of − 35 dB. The two elements of the antenna are rotated by 45° in the clockwise direction due to which resonance is shifted to 31 GHz with an isolation of − 15.5 dB as shown in Fig. 4b. The slots in the radiator or ground plane increase the effective inductance of the antenna while capacitance has negatively correlated with sloth width^3^. Therefore, the resonance frequency is reduced to 29 GHz by etching slots in the radiators. As seen in Fig. 4c, compared to the straight elements design, an improvement of − 20 dB was achieved in the mutual coupling for our proposed design. S_21_ was changed to − 36 dB at the resonance frequency of 29 GHz.

The scattering parameters of the presented MIMO antenna were measured in an anechoic chamber. Satisfactory results were observed for both simulated and measured results, as presented in Fig. 4c. The measured impedance bandwidth was higher than 1 GHz, which is sufficient for 5G communication devices. Similarly, the measured S_21_ was less than -32 dB at the resonance frequency.

**Figure 5:**
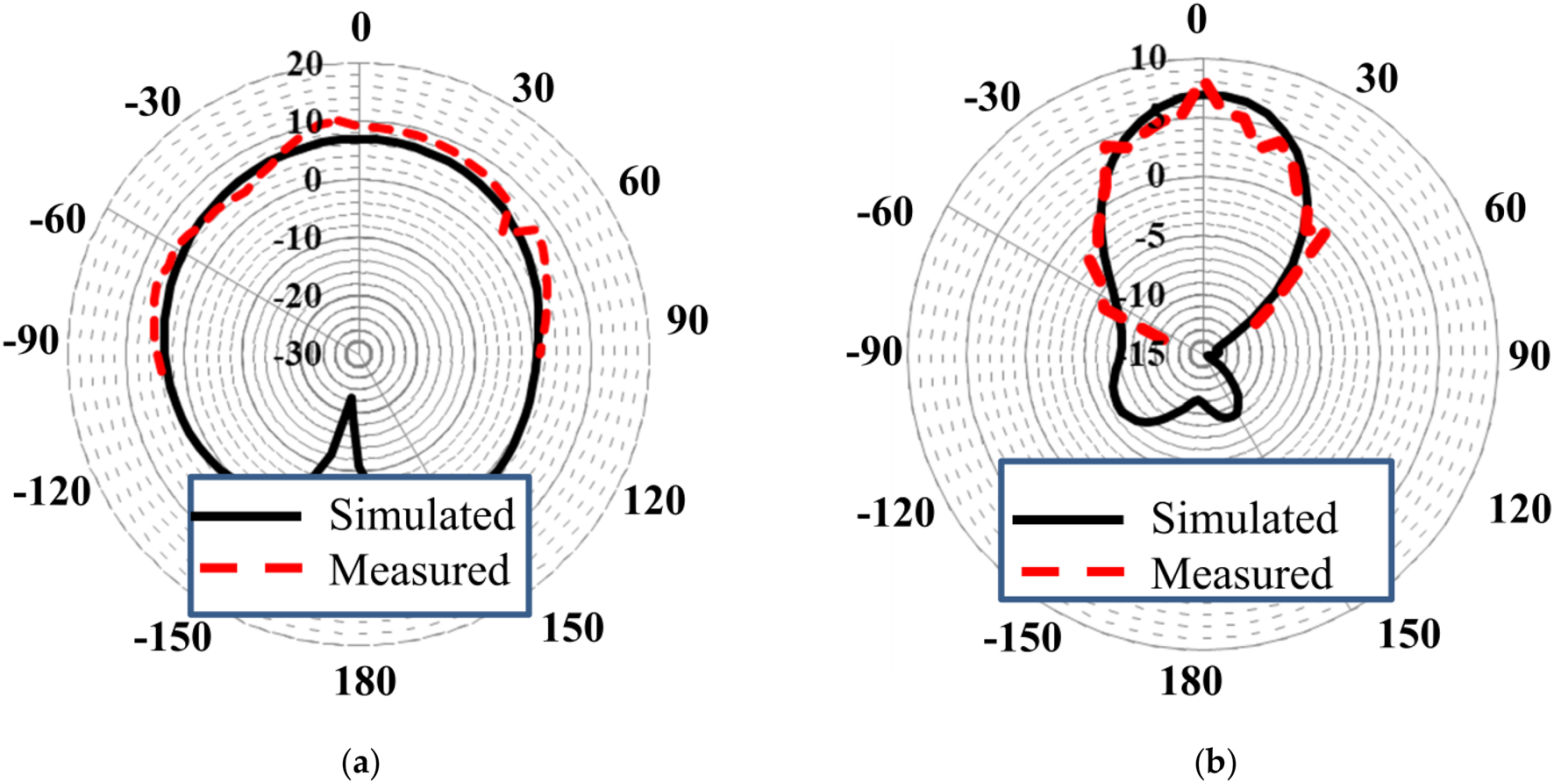
Radiation patterns in the (**a**) E-plane and (**b**) H-plane.

**Figure 6:**
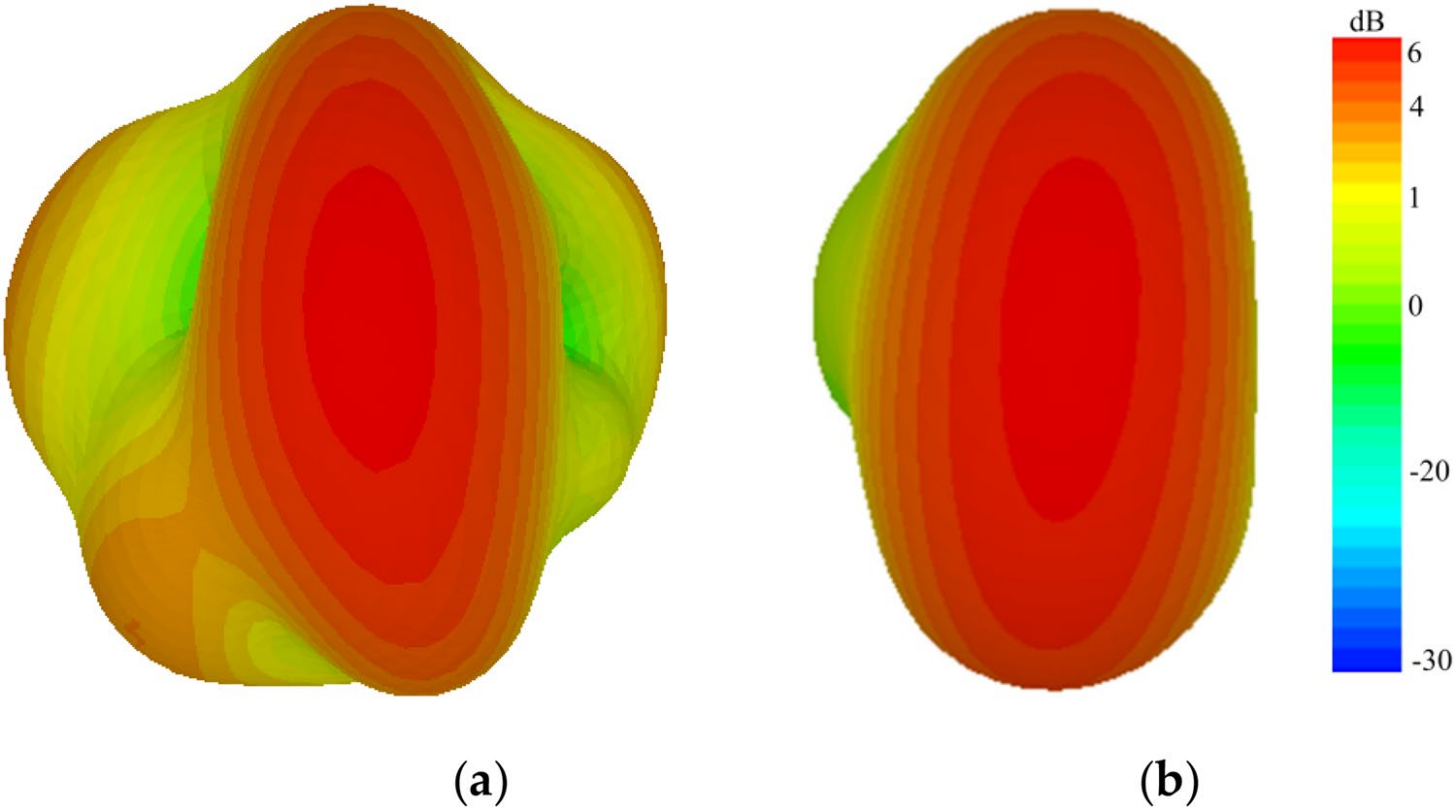
3D Radiation patterns (**a**) Simple MIMO antenna (**b**) Proposed MIMO antenna.

A close agreement was observed between the measured and simulated radiation patterns, as presented in Fig. 5a,b. Radiation patterns were measured with one port excited with 50-Ω feed while terminating the second one by a matching load. According to measurements, maximum gains of 6 and 5 dBi were observed in the E (xz)- and H (yz)-planes. Radiation patterns of the proposed antenna have very small back-lobe radiation, which increases its effectiveness for futuristic applications. The three-dimensional (3D) radiation patterns of the straight-element MIMO and proposed MIMO antenna for 29 GHz are presented in Fig. 6a,b, respectively. High side and back lobs were observed for the straight element MIMO antenna. However, by introducing slots in the radiating patch, main beam is directed towards a specific boresight direction with minimum sides and back lobes, thus making the proposed design efficient for 5G communication.

To validate the compatibility, performance, and efficiency of the proposed millimeter-wave MIMO antenna, envelope correlation coefficient (ECC) was investigated. The ECC is the relation between the incoming signals at the port of the antenna, and it is necessary to calculate the channel quality of the uncorrelated channel. A high ECC value leads to have high correlation and minimum isolation, which degrades antenna performance. Furthermore, ECC shows the mutual effect of the overall S-parameters of the designed MIMO antennas. The ECC can be found using different approaches, i.e., using the received signal envelope or by using S-parameter or far field radiation for estimation of complex cross correlation. In this paper, ECC is calculated by using both S-parameter and far field radiation patterns method as shown in Eqs. (3) and (4), respectively^24^. While for the measurements, the ECC from radiation patterns, involve a time consuming integral calculation. To ease the measurement process, ECC value of the proposed MIMO antenna system were calculated based on S-parameters using Eq. (3)

3 $$ECC=\frac{{\left|{S}_{ii}*{S}_{ij}+{S}_{ji}*{S}_{jj}\right|}^{2}}{\left(1-{\left|{S}_{ii}\right|}^{2}-{\left|{S}_{ji}\right|}^{2}\right)\left(1-{\left|{S}_{jj}\right|}^{2}-{\left|{S}_{ji}\right|}^{2}\right)}$$

4 $$\rho_{ij} = \frac{{\left| {\int {\int_{0}^{4\pi } {\left[ {\overrightarrow {{F_{i} }} \left( {\theta, \upphi } \right) \times \overrightarrow {{F_{j} }} \left( {\theta, \upphi } \right)d\Omega } \right]} } } \right|^{2} }}{{\int {\int_{0}^{4\pi } {\left| {\overrightarrow {{F_{i} }} \left( {\theta, \upphi } \right)} \right|^{2} d\Omega } } \int {\int_{0}^{4\pi } {\left| {\overrightarrow {{F_{j} }} \left( {\theta, \upphi } \right)} \right|^{2} d\Omega } } }}$$

where, ${\uprho }_{{{\text{ij}}}}$, represent the ECC. $\overrightarrow {{{\text{F}}_{{\text{i}}} }} \left( {\uptheta ,\upphi } \right)$ and $\overrightarrow {{{\text{F}}_{{\text{j}}} }} \left( {\uptheta ,\upphi } \right)$ are the radiation patterns of the i-th and j-th elements of the MIMO antenna, where I, j = 1, 2 for the proposed design.

**Figure 7:**
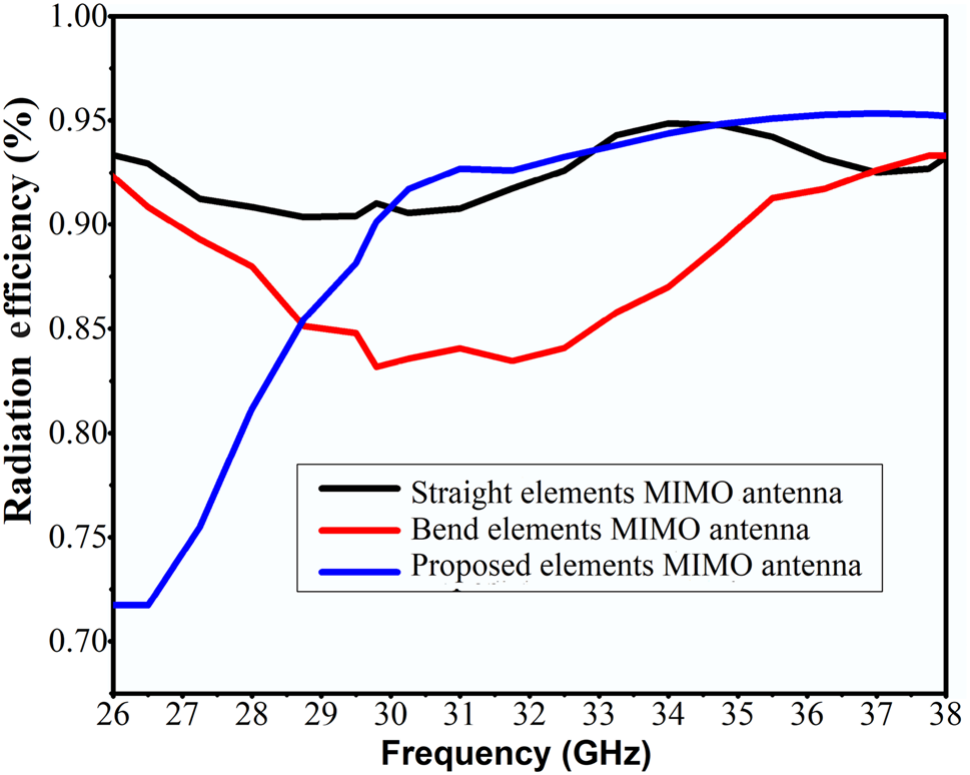
Simulated radiation efficiency of straight elements, bend elements, and proposed elements MIMO antenna.

**Figure 8:**
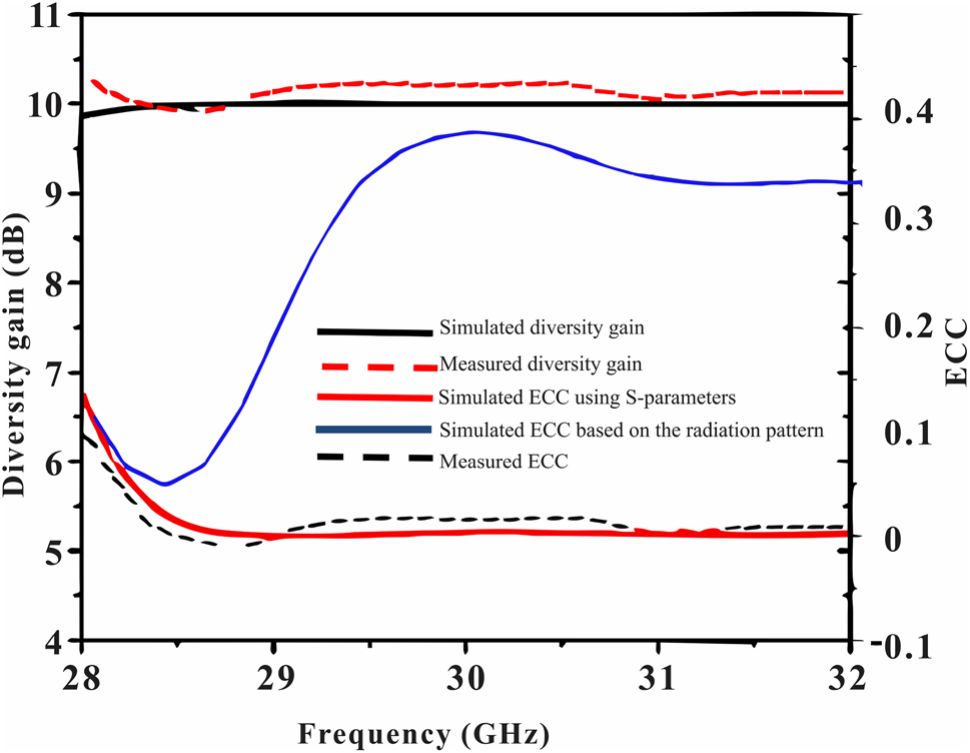
Simulated and measured ECC and diversity gain of the proposed antenna.

Radiation efficiency of straight elements, bend elements array and proposed elements MIMO antenna are given in Fig. 7. More than 85% radiation efficiency is realized for the proposed MIMO antenna across the operating band. Ideally, the ECC value should be zero. However, in practice, a limit of the ECC < 0.5 is adopted for uncorrelated MIMO^25^. Figure 8. presents that the proposed MIMO antenna has an ECC value of 0.0001 at the desired band of 29 GHz, which is considerably close to zero.

Diversity gain (DG) is also an important parameter that must be considered when evaluating the performance of MIMO antennas. This parameter is an indicative of the reliability of the MIMO system. Isolation of the radiators is higher for high DG antenna systems^26^. The following relation can be uses for calculating DG of the MIMO antenna:

5 $$DG=10\sqrt{1-{\left(ECC\right)}^{2}}$$

Diversity gain (DG) of the presented MIMO antenna is given in Fig. 8 in a range of frequencies. The presented antenna has a DG > 9.8 dB across the operating band.

**Figure 9:**
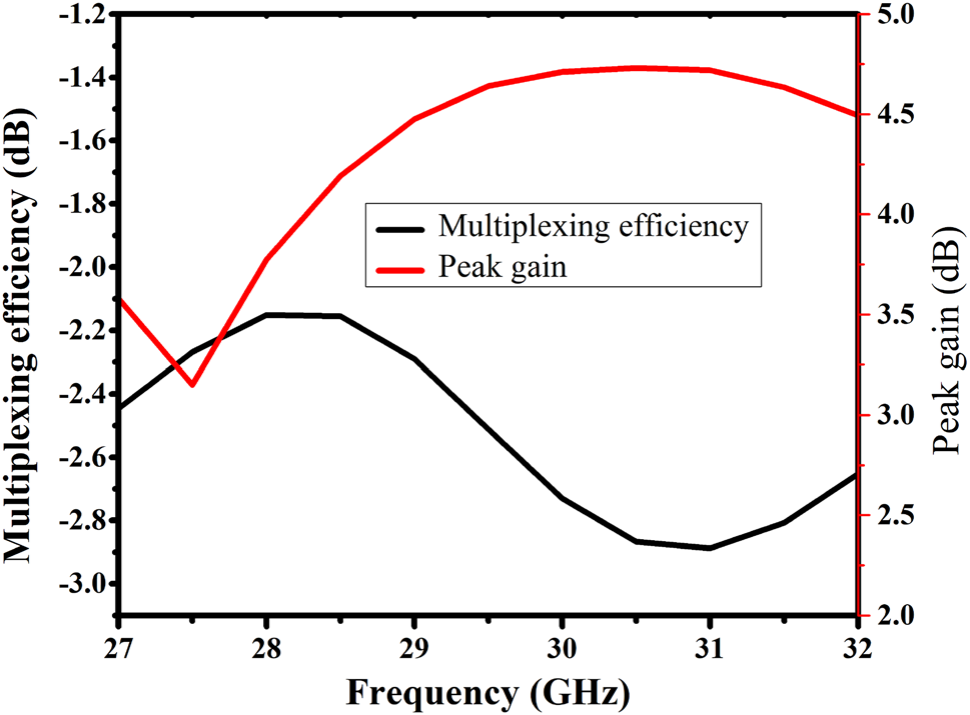
Simulated peak gain and multiplexing efficiency of the reported MIMO antenna.

Figure 9 shows multiplexing efficiency and peak gain with varying frequencies. Multiplexing efficiency (η_Mux_) is calculated by using the following relation:

6 $${\eta }_{Mux}=\sqrt{1-{\left|{\rho }_{c}\right|}^{2}{\eta }_{1}{\eta }_{2}}$$

where ‘${\uprho }_{\mathrm{c}}$’ is the complex correlation coefficient between the antenna patches. $\mathrm{ECC}\approx {\left|{\uprho }_{\mathrm{c}}\right|}^{2}$, and ${\upeta }_{\mathrm{i}}$ is the total efficiency of *i*-th antenna radiator. Here we used i = 1, 2 as we have two elements MIMO array.

Table 1 presents the comparison of the proposed with some recently published work based on different performance characteristics. In^27^, a millimeter-wave eight-element MIMO antenna array was proposed using beam tilting and high-isolation dielectric resonator. A minimum isolation of 25 dB and a peak gain of 7 dBi were achieved at the cost of large size and fabrication complexity. Similarly, Yagi–Uda antennas for a millimeter-wave MIMO terminal were presented in^28^, in which the proposed design achieved a better isolation of 21 dB; a horn-shaped Via was used for the isolation enhancement. A metasurface-based corrugation was used for mutual coupling reduction in^29^, and a better isolation of 37.1 dB was observed at the desired center frequency of 28 GHz. However, this design has a complex fabrication process. Similarly, in^30^, a four-port MIMO antenna was designed for millimeter-wave applications, providing better impedance bandwidth but achieving an isolation of only 21 dB with a wide dimension of 48 × 31 mm^2^. In^31^, quad-elements MIMO antenna is proposed for UWB applications. By using parasitic elements isolation is improve by 20 dB. In^32^, four elements MIMO antenna is proposed operating from 23 to 28 GHz, isolation is improve by using defective ground plane.

**Table 1:** Performance comparison with recent state of the art in the literature.

Refs.	Dim. (λo)	Technique used	No. of ports	Center frequency (GHz)	Fractional bandwidth (%)	Min. isolation (dB)	Edge to edge (λo)	Gain (dBi)
^27^	4.8 × 2.1	cDRA	2	30	6.1	25	λo/3.6	6.9, 8.6
^28^	NA	Horn-shaped Via	4	36	11	21	λo/1.27	10.8
^29^	3.9 × 7.9	Metasurface	2	28	17.8	37.1	NA	16.13
^30^	4.47 × 0.89	corner trimming	4	28	17	21	λo/6	10
^31^	0.41 × 0.44	Adding Decoupling structure	4	3.1	136	20	NA	4
^32^	1.85 × 5.6	DGS	4	25	22	28	NA	7.1
^14^	0.704 × 1.05	Wilkinson Power Divider	2	2.1	9.60	< 20	0.14 λo	NA
^5^	0.94 × 4.46	Near-Field Resonators	8	2.35	1.4	< 20	0.016 λo	6.8
Prop. work	1 × 0.5	Slits in the radiating part	2	29	3.7	36	λo/9	6

The proposed MIMO antenna maintained a compact structure of 11.7 × 5.3 mm^2^, due to the use of slotted and inclined antenna radiators. A better isolation of 36 dB, low ECC values of 0.0001, and high diversity gain of > 9.8 dB were achieved. A close agreement was found between both measured and simulated results, thereby making the proposed design a strong candidate for 5G millimeter-wave communications. The slight difference in measured and simulation results is due to fabrication tolerances.

## Conclusions

A compact 2 × 2 MIMO antenna with a shared ground plane was proposed in this study for futuristic 5G communication. Despite the simple geometry, a large reduction in antenna size (11.4 × 5.3 mm^2^) was achieved by introducing slits in the radiators to increase the current path. High isolation between the resonators was obtained by introducing slots in the patches and rotating them by 45° in the clockwise direction. The simulated results for ECC (ECC < 0.0001), DG (DG > 9.8 dB), and isolation between the radiators (> 36 dB) indicate that the presented MIMO antenna is an efficient candidate for 5G applications. The simulated and experimental results showed good agreement in term of S-parameters, radiation pattern, diversity gain and ECC, despite its miniaturized size in comparison with previous 29 GHz millimeter-wave antennas.
